# What factors influence MOOC course completion? An investigation of course completion and workplace benefits from interpersonal attraction theory perspective

**DOI:** 10.3389/fpsyg.2022.1055108

**Published:** 2022-11-22

**Authors:** Guihua Zhang, Dae-wan Kim, Jie Qi, Chenwei Zhao

**Affiliations:** ^1^Department of Business, Yeungnam University, Gyeongsan, South Korea; ^2^School of Business, Yeungnam University, Gyeongsan, South Korea; ^3^Department of Sociology, Yeungnam University, Gyeongsan, South Korea; ^4^Department of Korean Language Education as a Second Language, Yeungnam University, Gyeongsan, South Korea

**Keywords:** course completion, workplace benefits, thirst for knowledge, parasocial relationship, interpersonal attraction theory

## Abstract

MOOCs have attracted millions of learners worldwide by providing the public with convenient access to learning and quality educational resources, but the high dropout rate in MOOCs is still an urgent problem. Drawing upon Situation-Organism-Behavior-Consequence framework and interpersonal attraction theory, this study investigates the relationship between instructor attractiveness and MOOC course completion and further analyzes the impact of MOOC course completion on their career development. The results reveal that knowledge attractiveness and communication attractiveness significantly influence learners’ thirst for knowledge. Communication attractiveness and physical attractiveness significantly influence learners’ parasocial relationships. The thirst for knowledge and parasocial relationships are important antecedents of course completion for in-service learners and in-service learners’ completion of MOOC courses positively affects their workplace benefits. The findings provide new perspectives for the research domain of online education.

## Introduction

Online education is seen as an innovative way of teaching and learning, providing the public with a convenient way to learn and quality educational resources. With the development of online education, massive open online courses (MOOCs) have emerged. MOOC courses are larger in scale than traditional online education because they cater to the interests of learners, and people can participate in learning anytime and anywhere for free *via* the Internet as long as they are interested (Ma and Lee, 2019). MOOCs have attracted millions of learners worldwide and are considered an excellent medium for promoting lifelong learning (Meet and Kala, 2021) and have gained widespread public attention in the last few years (Yousef et al., 2014).

MOOC platforms bring many benefits to learning by offering courses for free, stimulating interest and enhancing the user experience, but high dropout rates in MOOCs are still the most common problem with the service (Jordan, 2015; Handoko et al., 2019). It has been revealed that the course completion rate for MOOCs is currently only between 5 and 40% (Bezerra and Silva, 2017). Improving course completion rates in MOOCs has been a hot issue for research. From the perspective of individual characteristics of learners, there is a significant link between different preferences of learners for course activities and MOOC course completion. For example, the more videos learners watch per week and the more articles and comments they publish per week, the higher the completion rate (Pursel et al., 2016; Del Peral Pérez, 2019). In addition, the task interest, time management, self-efficacy, and goal orientation of learners also significantly affect course completion (Wang and Baker, 2018; Handoko et al., 2019). However, from the current research, these factors are limited in increasing course completion rates, with one study suggesting that despite increased incentives for MOOCs, rates remain below 14% (Gil-Jaurena et al., 2017).

The interaction between instructors and learners and the quality of the videos provided have been emphasized as important factors in determining the completion of courses in MOOC (Gregori et al., 2018). A recent study confirms the importance of teachers in MOOC forums, where teachers play an active mediating role in the social cognitive knowledge construction of groups and improve learners’ performance (Liu et al., 2022). However, there is currently a lack of research on completion rates from the perspective of MOOC instructors (Gregori et al., 2018). In contrast, in traditional online education, the interpersonal attraction of the instructor can positively influence the relationship between instructor and learner, and the closer the relationship, the higher the learner engagement (Chen et al., 2021a,b). Hence, is there a connection between the interpersonal attractiveness of instructors and learner course completion in a MOOC? Verifying this will help inform MOOC operations and further improve course completion.

Many studies on MOOC completion rates are currently conducted on all MOOC users, and most assessments are limited to education-related sectors (such as learners and staff at universities); however, in reality, the motivation for participating in MOOCs often coincides with changing careers (Sablina et al., 2018). This may indicate that increasing the completion rate of MOOC courses is of great significance to the working population of non-education-related departments. Workplace people completing MOOC courses can increase not only knowledge but also accumulate capital to adapt to career development (Song and Jyung, 2018). Therefore, it is necessary to pay attention to the course completion of the on-the-job population. This has important implications for the operation of MOOCs. In connection with the interpersonal attractiveness of faculty presented above, this study examines the following research questions:

RQ1: What is the relationship between instructor attractiveness and course completion for the on-the-job population in a MOOC?To answer these questions, we propose a research model based on the SOBC framework and interpersonal attraction theory. In addition, a questionnaire survey on the target population is conducted, and the survey data is analyzed to clarify the relationship between instructor attraction, MOOC course completion of the working population, and organizational learning culture. This study will help MOOC platforms to develop operational plans for the working population and help the working population to adjust their learning plans to gain workplace benefits.

## Theoretical background and hypothesis development

### Interpersonal attraction theory

Originally proposed by Byrne and Griffitt (1973), interpersonal attraction theory was developed from social exchange theory. Furthermore, it seeks to explain the reason and development process of interpersonal relationships (Berscheid and Hatfield, 1969). The theory states that individuals evaluate other individuals’ abilities, physical attributes, specific behaviors, and emotional expressions constitute interpersonal attraction (Harris et al., 2003; Finkel and Baumeister, 2010) and such evaluations are generally positive and are the result of positive emotions, reactions, and behaviors of individuals toward other individuals (Montoya and Hibbard, 2014).

McCroskey et al. (1974) proposed that interpersonal attraction can be further subdivided into three dimensions: task, social, and physical attraction. First, interpersonal attraction is related to an individual’s ability to perform tasks and is a positive judgment of an individual’s ability to complete tasks (Connelly et al., 1997; Fang, 2014). Second, social attraction is an individual’s positive judgment of another’s ability to interact socially (Fang, 2014). Third, physical attraction is an individual’s positive judgment of another’s appearance and image (Berscheid and Hatfield, 1969).

In general, interpersonal attraction arises from an individual’s ability to provide economic, social, and resource benefits to another individual. In offline teaching practice, teachers with rich teaching experience or good image are more likely to improve students’ performance and more popular with students (Brooks and Young, 2015). In distance education, attraction positively influences the teacher-student relationship between the instructor and the student, the closer the relationship, the higher the student engagement (Chen et al., 2021a, 2021b). Nonetheless, the relationship between instructors’ interpersonal attractiveness and learners’ course performance in online education remains unclear and needs to be clarified.

### Situation-organism-behavior-consequence framework

Early research suggested that human behavior could be predicted and controlled through three stages: antecedent cues, behavior, and consequence, but such a process ignored the mediating role of individual cognition. In explaining mechanisms of self-behavior management, Luthans and Davis (1979) used the stimulus-organism-response framework (SOR) and antecedent-behavior-consequence model (ABC) as the basis for proposing the Situation-Organism-Behavior-Consequence framework (SOBC). First, SOBC extends the ABC model to include cognitive processes (O), suggesting that cognitive processes have a mediating role between stimuli (S) and consequences (C). Second, SOBC extended the SOR model to include the consequences (C) resulting from the act of taking (Dhir et al., 2021). This resulted in the four components of the SOBC framework: stimulus, organism, behavior, and consequence. These begin with the identification of the stimulus and then derive individual behaviors and possible outcomes based on the organism’s response to the stimulus.

In education, learners’ learning tendencies are stimulated by the instructor’s style and change (Hidayah et al., 2017; Husin et al., 2021). Learner responses to instructor stimuli in a course have important implications for course performance, such as the sequence and structure of course delivery. It has been noted that when instructors provide stimuli in the form of a task, learners reflect, make a general assessment of the task, and then respond (Hidayah et al., 2017). For example, excellent teaching content can provide positive stimuli to learners and develop their questioning and thinking skills, thus improving their learning performance (Hidayah et al., 2017). Therefore, when evaluating course effectiveness, it is necessary to examine specific educational scenarios in terms of classroom stimuli, learner assessment processes, learning behaviors, and final performance. The SOBC framework provides a powerful and structured mechanism to explain the role of learner experience, self-regulation, and performance in a variety of educational settings (Whelan et al., 2020).

#### Stimulus and organism in MOOC

The stimulus in the environment is explicit or implicit. It induces an internal state in the individual to drive certain behaviors (Whelan et al., 2020). A study on social commerce revealed that attractiveness could significantly stimulate consumers’ willingness to purchase on online platforms (Liu et al., 2018), and such attractiveness can also stimulate consumers and media to build more intimate relationships (Zheng et al., 2020). Such relationships can be extended to MOOCs; Teacher attraction not only stimulates student learning, but also creates a good teacher-student relationship. The organism is the mediated state of the user’s cognition and emotion, which is reflective of the process between the stimulus and the user’s response (Jacoby, 2002). The cognitive state represents the user’s mental processes, including eagerness, satisfaction, uneasiness, panic, and so on. Emotional states reflect the user’s excitement and hedonic or fatigue emotions after being stimulated by the environment. Once exposed to the stimulus, users process the stimulus information, and this helps them make decisions (Islam and Rahman, 2017).

This study focuses on the thirst for knowledge and parasocial relationships generated by the organism in MOOCs. The thirst for knowledge is curiosity that stimulates investigation and learning, a curiosity for knowledge, a desire for knowledge, and an active state of learning (Jensen, 2012). Learners may notice that they do not have sufficient knowledge to solve problems, and they use online education platforms to learn more (Golman and Loewenstein, 2018), or they may be stimulated by education platforms and have a strong thirst for knowledge. Emotional bonds inevitably form between viewers and media personalities in online platforms, creating parasocial relationships (Hu et al., 2020), which can lead to positive perceptions and increased user loyalty, promoting user engagement (Redd, 2012; Labrecque, 2014). MOOC is a web-based education where learners interact with instructors by watching videos, so it is inevitable that a parasocial relationship, one of the most common emotions among learners using online education platforms, also arises between them.

Task attraction in interpersonal attraction refers to an individual’s positive judgment of another person’s ability to complete a task (McCroskey and McCain, 1974). In education, it is most important for instructors to have considerable expertise and teaching experience to accomplish their teaching tasks (Siedentop and Eldar, 1989), and for learners to acquire some expertise through instructors to accomplish their learning tasks (Connelly et al., 1997). In MOOCs, instructors will try to impart professional knowledge to users to complete teaching tasks, and users may have a thirst for unknown knowledge due to the coherence of knowledge acquisition on the one hand, and a thirst for knowledge on the other as they may feel that learning more knowledge can complete their learning tasks (Liang et al., 2011). In MOOCs, if users feel that actively participating in the courses of these college instructors can gain professional knowledge, help make correct decisions, and improve performance, users would thank the instructors. On the other hand, there are many famous instructors in MOOCs, and users follow them with admiration, hoping to learn more knowledge from their courses. Both gratitude and admiration are positive emotions exhibited by users, which are helpful in establishing parasocial relationships in MOOC (Xiang et al., 2014). Therefore, we propose the following hypotheses:

*H1a*: In MOOCs, the instructor’s knowledge attractiveness has a positive impact on in-service learners’ thirst for knowledge*.*

*H1b*: In MOOCs, the instructor’s knowledge attractiveness has a positive impact on the establishment of parasocial relationships*.*

Social appeal is the degree to which a website or system can facilitate social interaction with individuals (Preece, 2001). Social competence is expressed in teaching mainly through communication between teachers and students, and communication competence and social competence are closely related (Jurevičienė et al., 2012). The stronger the communication skills in teaching, the stronger the social skills, which can reflect the teacher’s social-based interpersonal attractiveness. A social appeal is a key feature of online platforms where users can enjoy interacting with others to gain shared information and benefits (Croes et al., 2018). Communication is the core of social life, and social and communication skills are closely related (Jurevičienė et al., 2012). The real goal of teaching is the acquisition of knowledge by developing learners’ intellect, stimulating their curiosity and knowledge-seeking, and stimulating their abilities (Suprapto et al., 2020). Thus, communicating tactfully can make the classroom atmosphere more comfortable. In online education platforms, good communication can mean triggering users’ desire to learn and building parasocial relationships. On the one hand, communication helps instructors to better transfer knowledge to users, and good communication may make users curious about future knowledge, and their thirst for knowledge will be stronger. On the other hand, the desire for knowledge inspired by communication may lead to feelings of admiration, which in turn leads to more communication between users and teachers. This means that good communication helps to establish close quasi-social relationships with others more efficiently (Lin et al., 2021). Therefore, we propose the following hypotheses:

*H2a*: In MOOCs, the instructor’s communicative attractiveness has a positive impact on in-service learners’ thirst for knowledge*.*

*H2b*: In MOOCs, the instructor’s communicative attractiveness has a positive effect on the establishment of prosocial relationships*.*

Physical attractiveness is an individual’s positive judgment of another person’s appearance and image (Huston and Levinger, 1978). An attractive appearance and image may be an asset, and physical attractiveness is an important clue for evaluating a person (Kim and Yang, 2021). Physical attractiveness has been used in marketing to explain the effectiveness of celebrity endorsements, and studies have found that having an attractive appearance and image improves their social skills (Till and Busler, 2000). In education, instructors with a good image are more popular with learners (Brooks and Young, 2015). A good image helps win the favor and trust of learners, generates positive emotions, and continuously stimulates learners’ thirst for knowledge while also establishing a harmonious instructor-learner relationship, which is a parasocial relationship when placed in a MOOC based on network operations. Therefore, we propose the following hypotheses:

*H3a*: In MOOCs, the instructor’s physical attractiveness has a positive effect on in-service learners' thirst for knowledge*.*

*H3b*: In MOOCs, the instructor’s physical attractiveness has a positive effect on the establishment of parasocial relationships*.*

#### Organism and behavior in MOOC

The main behavior that learners display after the organism is stimulated is to try to complete the course, and course completion is one of the most important behaviors of MOOC learners (Handoko et al., 2019).

Thirst for knowledge means to stimulate the curiosity of investigation and learning, which is a state of being curious about knowledge, thirst for knowledge, eager to learn, and wanting to know more. The desire to learn is a dynamic and continuous action (Jensen, 2012). A learner who is thirsty for knowledge may ask more questions or search for useful information on the platform when they find that they do not have sufficient knowledge to solve the problem (Golman and Loewenstein, 2018). Thirst for knowledge has a direct impact on learning and can enable learners to continue to participate in learning. When learners are motivated to seek knowledge to fill knowledge gaps, they are likely to learn more (Lamnina and Chase, 2019). In MOOCs, thirst for knowledge predicts many positive outcomes, such as knowledge management (Lai et al., 2019) and academic performance (Khuram et al., 2021). Therefore, we propose the following hypothesis:

*H4a*: In MOOC, in-service learners’ thirst for knowledge has a positive impact on their course completion*.*

Parasocial relationships refer to the emotional bonds formed between viewers and media personalities and are one-way relationships (Hu et al., 2020). Previous research has shown that such relationships can lead to positive perceptions and increased user loyalty (Labrecque, 2014). These relationships are always a positive factor for social media users, and they greatly facilitate user engagement (Redd, 2012). Parasocial relationships during live streaming can draw users back into the content and even translate into an emotional connection with the media persona (Quintero Johnson and Patnoe-Woodley, 2016). In MOOCs, in-service learners will trust the instructor more because of the parasocial relationship they have established. Moreover, they may be guided step-by-step by instructors, and will be more likely to complete the course. Therefore, we propose the following hypothesis:

*H4b*: In MOOC, the parasocial relationship between in-service learners and the instructor has a positive impact on their course completion*.*

#### Behavior and consequences in MOOC

In-service learners may reap workplace benefits upon completion of the program, and to accumulate capital to adapt to career development (Song and Jyung, 2018), workers will strive to complete the courses in MOOC. MOOCs provide proof of course (Liu et al., 2021), and many can use the proof to advance their careers (Banks and Meinert, 2016). A survey of more than 50,000 people noted that 72% of respondents had gained professional benefits from using a MOOC, 52% had improved their workplace lives, and respondents reported that participating in a MOOC had led to a promotion in their previous job or a new job that suited them better (Zheng et al., 2015). Therefore, we propose the following hypothesis:

*H5*: In MOOCs, in-service learners complete courses that positively affect their workplace benefits*.*

## Research model and method

### Research model

**Figure 1:**
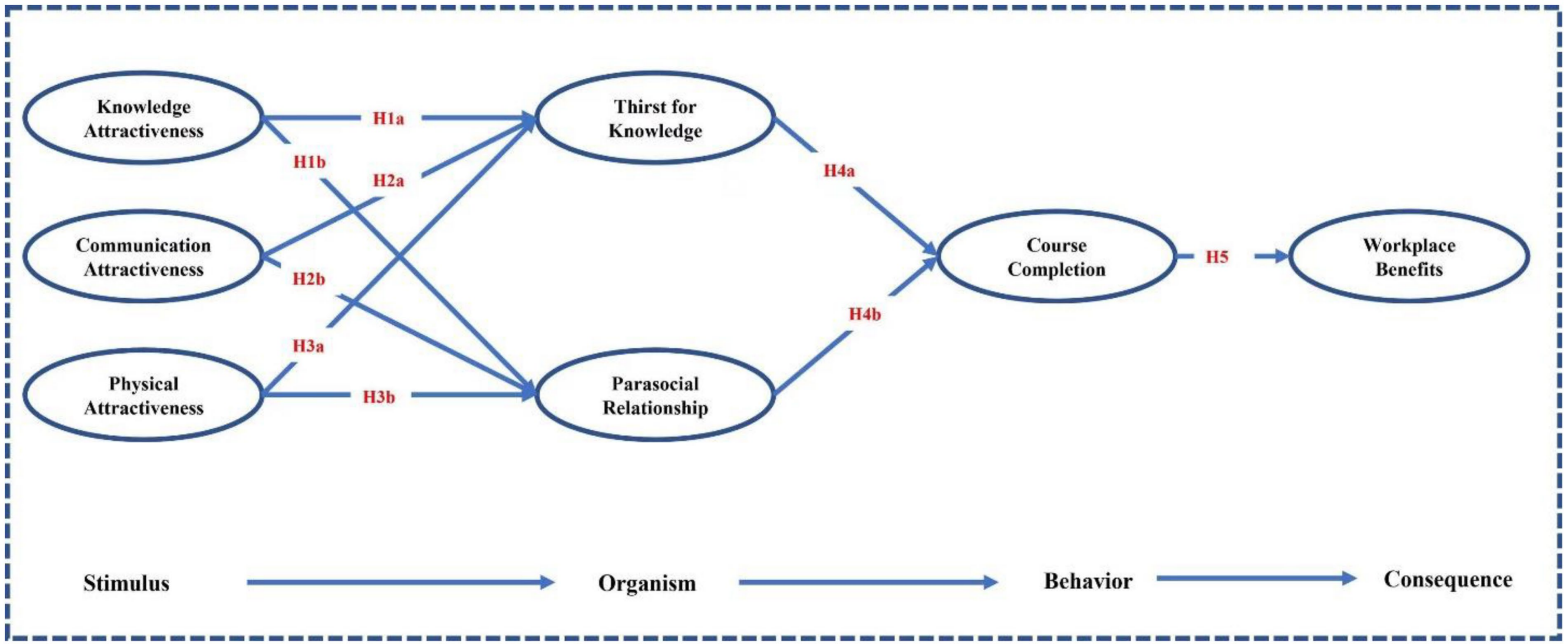
Proposed research model.

The research method is illustrated in Figure 1. This study established a model based on the SOBC framework and instructor attractiveness. In this model, the instructors in MOOC stimulate in-service learners’ thirst for knowledge and the establishment of parasocial relationships through knowledge attraction, communication attraction, and physical attraction. These two positive organismic emotions guide the in-service learners to complete the course. Course completion enables in-service learners to acquire new knowledge and skills that will help them gain workplace benefits (e.g., increased income, job advancement, etc.).

### Method

We designed a questionnaire based on existing studies, combined with the actual situation of this study. We used a 5-point Likert scale (1 = “very disagree” to 5 = “very agree”) to measure the questionnaire items, and the questions were based on those validated in existing studies and modified to fit the context of this study. After the questionnaire was completed, it was reviewed by experts in the field and then 70 university students were invited to conduct a pretest to ensure the validity of the questionnaire. The final questionnaire can be found in Appendix A.

The questionnaire was distributed online through social networks. Before doing so, we consulted the academic ethics committee of the authors to ensure that the questionnaires did not involve ethical issues. All participants were provided with the following information: (1) the questionnaire was anonymous, (2) the content and purpose of the survey were clearly stated, (3) all participants were free to answer or not to answer the questionnaire, (4) the questionnaire did not involve personal or private information, and (5) all participants would receive a gift after completing the questionnaire.

According to the Baidu index,^1^ the Jiangsu region of China has the highest level of interest in MOOCs, so we conducted a survey on people in the workplace in Nanjing, Jiangsu Province, China. To avoid collecting inaccurate data, we designed the questionnaire in such a way that all questions were mandatory. If an item was not answered, the questionnaire could not be submitted. If participants selected an item of “not using MOOC” or “not a workplace person,” the survey was automatically terminated and no follow-up survey was conducted. The survey period was from November 20 to December 20, 2021. A total of 598 responses were received, and invalid answers (duplicate responses, response time less than 2 min) were removed, leaving 306 valid responses. To avoid non-response bias, we conducted a paired t-test using the top and bottom 20 of the respondents. The results indicated that all demographic items were at the probability level of 0.05, and the differences between the two groups were not statistically significant.

After completing the survey, we first conducted a demographic analysis. Of the 306 participants, 175 (57.3%) were male, and 131 (42.7%) were female. The proportion of those aged 31 to 40 years was higher (*N* = 171, 55.9%), followed by those aged 21–30 years, with 72 (23.5%). A total of 54 (17.6%) respondents had no higher education, 131 (42.8%) had a bachelor’s degree, and 25 (8.2%) had a master or doctoral degree. Most participants (*N* = 205, 67%) had more than 5 years of work experience. Most respondents had a monthly income between 3,001 and 4,000 RMB (USD $441 to $558; *N* = 92, 30%), followed by those with a monthly income of 4,001–5,000 RMB (USD $588 to $735; *N* = 67, 22%).

## Data analyses and results

### Common method bias

In social science research, Harman’s univariate analysis has been widely used to estimate the likelihood of common method bias (Harman, 1976). Using this method to extract a single factor, if the variance is less than 40%, the survey data are minimally affected by the bias of the commonly used methods (Podsakoff et al., 2003). We conclude that it is not a problem in this study.

### Analysis method

This study employs the partial least squares structural equation modeling (PLS-SEM) method using SmartPLS 3.0. Although PLS-SEM is similar to regression analysis, it can measure both the validity and reliability of the variables, as well as the path relationship between the variables and the explanatory power of the model. Least squares can be used for prediction and it is less restrictive on sample size than other methods (Hair et al., 2012). PLS-SEM is more suitable for measuring complex models, especially those with more than six variables (Hair et al., 2012). Meanwhile, PLS-SEM allows the analysis of non-normally distributed data.

Firstly, this study included a total of eight variables. Secondly, the multivariate normal analysis of the sample data in this paper shows that Mardia’s multivariate skewness (β = 202.863, *p* < 0.01) and multivariate kurtosis (β = 549.057, *p* > 0.05), which suggests multivariate non-normality. Therefore, choosing PLS-SEM for data analysis was more suitable for this study.

### Measurement model

We employ measurement models to confirm the appropriate use of variable terms, including reliability and discriminant validity. As reported in Table 1, the factor loading values of all items were significantly higher than the benchmark value of 0.7. These results indicate that these structures exhibit good consistency (Hair et al., 2012). We used the composite reliability of the facets and the extracted mean–variance (AVE) to test for convergent validity. As presented in Table 2, AVE values ranged from 0.646 to 0.759, above the baseline value of 0.5. The combined reliability ranges from 0.844 to 0.902, thus achieving the recommended benchmark of 0.7 (Campbell and Fiske, 1959). Discriminant validity shows how the items differ from each other. Table 2 shows that the square root of AVE for all structures is higher than the correlation between different structures (Fornell and Larcker, 1981).

**Table 1:** Assessment of reliability and convergent validity.

Constructs	Item	Loading	Cronbach’s a	CR	AVE
KNA	KNA1	0.927	0.838	0.902	0.755
	KNA2	0.843			
	KNA3	0.835			
COA	COA1	0.920	0.826	0.896	0.743
	COA2	0.824			
	COA3	0.838			
PHA	PHA1	0.899	0.841	0.904	0.759
	PHA2	0.854			
	PHA3	0.860			
THK	THK1	0.914	0.728	0.844	0.646
	THK2	0.728			
	THK3	0.757			
PAR	PAR1	0.912	0.731	0.846	0.650
	PAR2	0.736			
	PAR3	0.758			
COC	COC1	0.920	0.733	0.849	0.653
	COC2	0.824			
	COC3	0.838			
WOB	WOB1	0.884	0.786	0.865	0.682
	WOB2	0.857			
	WOB3	0.728			

KNA, knowledge attractiveness; COA, communication attractiveness;, PHA, physical attractiveness; THK, thirst for knowledge;, PAR, parasocial relationship; COC, course completion; WOB, workplace benefits.

**Table 2:** Fornell-Larcker criterion test.

	KNA	COA	PHA	THK	PAR	COC	WOB
KNA	**0.869**						
COA	−0.064	**0.862**					
PHA	−0.046	−0.011	**0.871**				
THK	0.251	0.347	−0.052	**0.804**			
PAR	−0.113	0.376	0.334	0.565	**0.806**		
COC	0.026	0.215	−0.009	0.395	0.440	**0.808**	
WOB	0.070	0.018	−0.041	0.130	0.122	0.360	**0.826**

KNA, knowledge attractiveness; COA, communication attractiveness;, PHA, physical attractiveness; THK, thirst for knowledge;, PAR, parasocial relationship; COC, course completion; WOB, workplace benefits.

We tested the goodness-of-fit (GoF) of the model. The GoF (0 < GoF < 1) is defined as the geometric mean of the average communality and average R^2^ (for endogenous constructs)， GoFsmall = 0.1, GoFmedium = 0.25, and GoFlarge = 0.36 (Tenenhaus et al., 2005). The GoF of our model was calculated to be 0.284, indicating that the fit met the standard and that the model performed well. Finally, we tested the collinearity problem, and the VIF between all variables was below 5, indicating that there was no collinearity problem.

### Structural model

**Figure 2:**
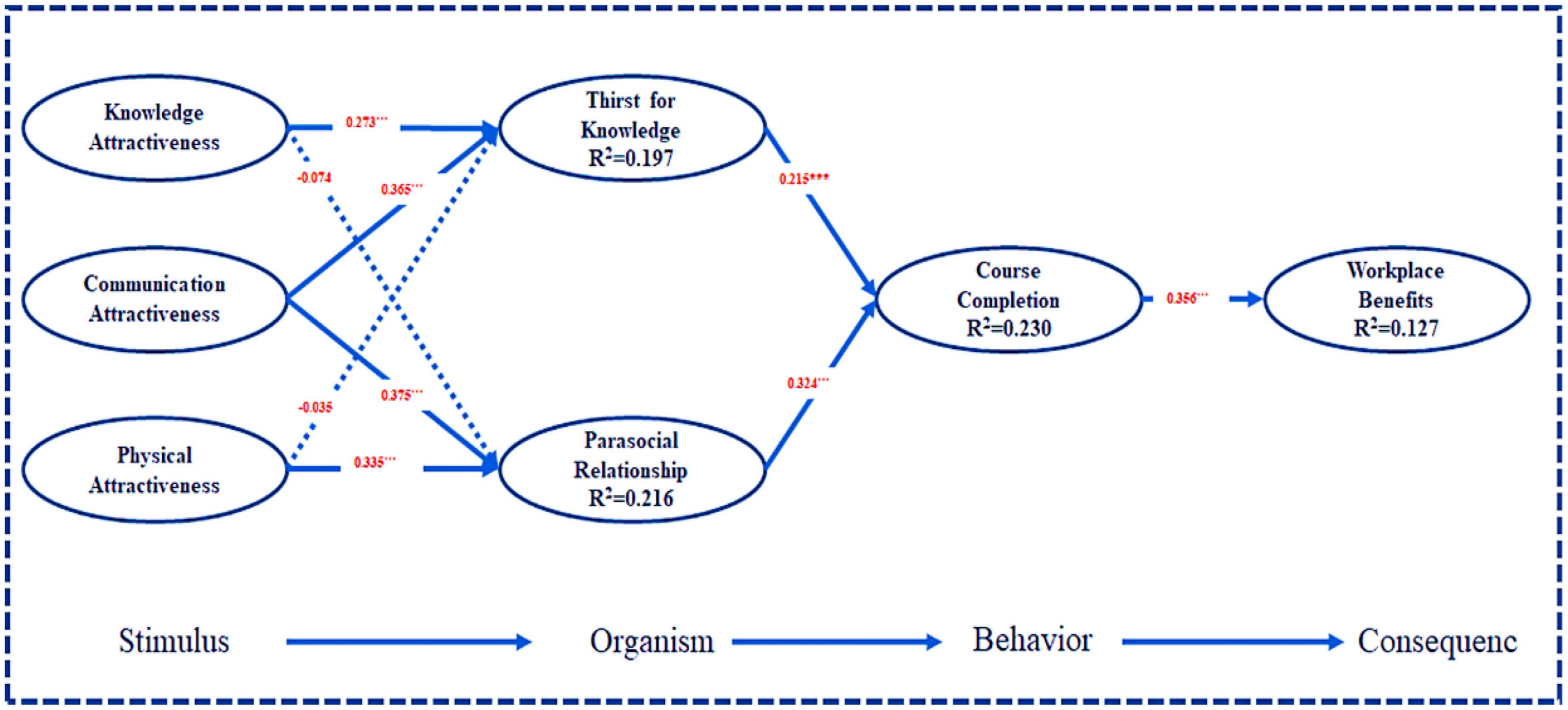
Test results of the structural model test. Note: ***p < 0.001, ** *p* < 0.05.

While testing the research model, we tested the overall explanatory power of the structural model, the amount of variance explained, and the strength of the path. The path coefficient was obtained using SmartPLS 3.0 (Figure 2). As presented in Table 3, there was a positive relationship between “Knowledge Attractiveness” and “Thirst for Knowledge” (β = 0.273, *p* < 0.001), suggesting that hypothesis H1a was supported. The relationship between “Knowledge Attractiveness” and “Parasocial Relationship” was not significant (ß = −0.074, ns), indicating that hypothesis H1b was not supported. There was a positive relationship between “Communication Attractiveness” and “Thirst for Knowledge” (β = 0.365, *p* < 0.001), suggesting that hypothesis H2a was supported. “Communication Attractiveness” was positively correlated with “Parasocial Relationship” (β = 0.375, *p* < 0.001), supporting hypothesis H2b. The relationship between “Attractiveness” and “Thirst for Knowledge” was not significant (ß = −0.035, ns), indicating that hypothesis H3a was not supported. There was a positive relationship between “Physical Attractiveness” and “Parasocial Relationship” (β = 0.335, *p* < 0.001), suggesting that hypothesis H3b was supported.

**Table 3:** Hypothesis testing results.

Hypotheses	ß	STDEV	T statistics	*p* values	Result
H1a: KNA - > THK	0.273	0.047	5.786	0.000	Support
H1b: KNA - > PAR	−0.074	0.050	1.469	0.142	Reject
H2a: COA - > THK	0.365	0.047	7.728	0.000	Support
H2b: COA - > PAR	0.375	0.044	8.426	0.000	Support
H3a: PHA - > THK	−0.035	0.051	0.681	0.492	Reject
H3b: PHA - > PAR	0.335	0.047	7.179	0.000	Support
H4a: THK - > COC	0.215	0.062	3.443	0.001	Support
H4b: PAR - > COC	0.324	0.060	5.355	0.000	Support
H5: COC - > WOB	0.356	0.045	7.970	0.000	Support

KNA, knowledge attractiveness; COA, communication attractiveness; PHA, physical attractiveness; THK, thirst for knowledge; PAR, parasocial relationship; COC, course completion; WOB, workplace benefits.

As reported in Table 3, there was a positive relationship between “Thirst for Knowledge” and “Course Completion” (β = 0.215, *p* < 0.001), suggesting that hypothesis H4a was supported. “Parasocial Relationship” was positively correlated with “Course Completion” (β = 0.324, *p* < 0.001), supporting hypothesis H4b. Finally, “Course Completion” was positively associated with “Workplace Benefits” (β = 0.356, *p* < 0.001), supporting hypothesis H5.

## Discussion and implications

### Discussion of key findings

There are several interesting findings of the study. First, knowledge attractiveness and communication attractiveness influenced in-service learners’ thirst for knowledge, but physical attractiveness did not have a significant direct effect. The results are consistent with previous studies that demonstrate that viewers prefer characters who are socially articulate and task completers over those who simply look good and have a good image (Zheng et al., 2020).

Second, communicative attractiveness and physical attractiveness significantly affected in-service learners’ para relationships with instructors, but knowledge attractiveness did not have a significant effect. One possible explanation is that the establishment of a parasocial relationship is predicated on the user’s attachment, admiration, and fantasy of the media personality, while the maintenance of such relationship requires the user’s continuous following (Hwang and Zhang, 2018). But mastering specialized knowledge is difficult and often requires a long-term commitment. In particular, when learners are not sufficiently grounded to understand advanced specialized vocabulary, they become frustrated, anxious, and thus unable to continue learning with the instructor, so the predictive power of knowledge attraction for establishing parasocial relationships is reduced.

Third, the results of our analysis of the drivers of course completion for in-service learners indicate that parasocial relationships and thirst for knowledge are important antecedents in predicting course completion. This is consistent with previous research findings that such relationships can help users achieve results (Chen et al., 2021a). The formation of parasocial relationships during the delivery of the course has a positive impact on the completion of the course by the in-service learners and increases the completion rate of the platform course. The thirst for knowledge is important in predicting the course completion behavior of online education users. It triggers individual information-seeking behavior that facilitates the construction of knowledge and helps learners complete the course.

Finally, the results indicate that the completion of the MOOC course has a positive impact on users’ career development. Some studies have suggested that working people can realize career development through the MOOC certificate obtained after studying in MOOC (Trumbore, 2021). Currently the only way to obtain a MOOC certificate is to complete the course, so our study confirms this result by demonstrating that user completion of a MOOC course positively affects the user’s workplace benefits.

### Theoretical and practical contributions

The results of the study give several theoretical and practical insights. First, most previous research on MOOC platforms has been learner-centered, with less research on instructors (Meet and Kala, 2021). From the perspective of instructors, this study takes attractiveness as the starting point, divides instructor attractiveness into three important dimensions: knowledge attractiveness, communication attractiveness, and physical attractiveness, and examines its influence on users’ perception and behavioral intentions. It provides a new theoretical basis for future research on the influence of MOOC instructors on the role of learning outcome management and platform governance.

Second, we analyzed MOOC user behavior based on a parasocial relationship perspective. Previous literature has considered technological environments and virtual experiences to stimulate sustained user use (Zhao et al., 2020), but lacks analysis of the relationship between instructors and learners. This study argues that the MOOC platform is a communication environment, and it is very important to consider interpersonal relationships among members. By introducing the concept of quasi-social relationships, an in-depth analysis of the instructor-learner relationship in the MOOC environment is conducted, expanding the literature on online education.

Finally, this study expands the literature on online education through an empirical investigation of the relationship between course completion and workplace benefits. Few previous studies have focused on MOOC learning outcomes to understand whether improved knowledge will improve learners’ employability or whether it will enhance their job positions (Meet and Kala, 2021). This study bridges this gap and provides a theoretical basis for subsequent studies.

This paper also has some practical contributions. First, teachers of educational platforms must be aware of the importance of teacher expertise and effective communication and practice it in their teaching process. Teachers need to improve their professional knowledge and teaching skills. A successful teacher must not only have a wealth of professional knowledge and expertise. It is also important to know about pedagogy and psychology. Language expression makes the most important basic skills of teachers, and teaching objectives should be concise to make teaching content as concise and easy to understand as possible. It is also recommended that teachers make more flexible use of multilateral interactions to guide students’ thinking and communication to achieve better teaching results.

Next, thirst for knowledge and parasocial relationships are important determinants of user course completion. Practitioners should pay sufficient attention to these aspects. Practitioners can consider actively guiding users to explore and seek knowledge by providing rewards. For example, higher credits and platform rewards can be awarded to learners who actively participate in course discussions and are expressive. The platform can establish a reward mechanism to stimulate users’ interest through points, badges, and rankings to increase their desire to explore and learn. The platform should encourage influential master teachers and experts to continuously introduce new hot topics, create an environment to inspire learners to actively seek knowledge, and maintain good parasocial relationships through Q&A interactions.

### Limitations and further directions

This study has some limitations. First, this study only focused on the perspective of instructor attractiveness when analyzing learners’ knowledge seeking and prosocial relationships. Many other factors still deserve to be considered and studied, and future research should analyze more stimulus factors.

Third, we used survey data to validate the relationships in the model; any online survey is self-selective in nature, and it is impossible to determine whether participants’ responses were biased. Meanwhile, the respondents were in-service learners using Chinese MOOCs, so the results are not representative of all users. Future research can use experimental or longitudinal methods to analyze user behavior and explore it in more detail.

## Data availability statement

The original contributions presented in the study are included in the article/supplementary material, further inquiries can be directed to the corresponding author.

## Author contributions

GZ and CZ: conceptualization. GZ: methodology and writing—original draft preparation. D-wK: software and project administration. GZ and JQ: formal analysis. GZ, JQ, and CZ: writing—review and editing. All authors contributed to the article and approved the submitted version.

## Conflict of interest

The authors declare that the research was conducted in the absence of any commercial or financial relationships that could be construed as a potential conflict of interest.

## Publisher’s note

All claims expressed in this article are solely those of the authors and do not necessarily represent those of their affiliated organizations, or those of the publisher, the editors and the reviewers. Any product that may be evaluated in this article, or claim that may be made by its manufacturer, is not guaranteed or endorsed by the publisher.

## Footnotes

^1^ http://index.baidu.com

## Appendix A

**Table:**

Factors	Items	Item	Reference
Knowledge attractiveness	KNA1	I pay more attention to the courses of instructors with professional knowledge.	Shen et al. (2010)
	KNA2	I will buy the course if the instructor is famous.	
	KNA3	I think a course taught by an instructor with expertise is more attractive.	
Communication attractiveness	COA1	I think instructors with good communication skills are more persuasive.	Shen et al. (2010)
	COA2	I would prefer to be involved in a class where the instructors interact with me on a regular basis.	
	COA3	I think instructors are good at communicating to make the course more interesting.	
Physical attractiveness	PHA1	MOOC’s instructors make me feel comfortable visually.	Zheng et al. (2020)
	PHA2	The course instructor seems friendly I will participate actively.	
	PHA3	I think a course led by an instructor with a good image is more attractive.	
Thirst for knowledge	THK1	In class, I want to know more about what the instructor has just said.	Lamnina and Chase (2019)
	THK2	In class, I want to find out more about what I have learned.	
	THK3	I want to acquire more knowledge and skills.	
Parasocial relationship	PAR1	I look forward to seeing the instructors on the platform.	Hu et al. (2020)
	PAR2	I will interact with the instructor on the platform.	
	PAR3	The class instructor makes me feel comfortable, like an old friend.	
Courses completed	COC1	I have listened to all the contents of the course.	Evans et al. (2016)
	COC2	I have completed all assignments and exams.	
	COC3	I have achieved the desired results and certificates.	
Workplace benefits	WOB1	Career skills acquired through MOOC help me achieve more benefits.	Haryono et al. (2020)
	WOB2	The knowledge and experience acquired through MOOC could potentially lead to my promotion.	
	WOB3	The career skills acquired through MOOC have enabled me to gain more development opportunities in the company.	
